# Corpus Callosum Size Is Highly Heritable in Humans, and May Reflect Distinct Genetic Influences on Ventral and Rostral Regions

**DOI:** 10.1371/journal.pone.0099980

**Published:** 2014-06-26

**Authors:** Girma Woldehawariat, Pedro E. Martinez, Peter Hauser, David M. Hoover, Wayne W. C. Drevets, Francis J. McMahon

**Affiliations:** 1 Genetic Basis of Mood & Anxiety Disorders Section, Human Genetics Branch, National Institute of Mental Health, NIH, DHHS, Bethesda, Maryland, United States of America; 2 Section on Behavioral Endocrinology, National Institute of Mental Health, NIH, DHHS, Bethesda, Maryland, Unites States of America; 3 VISN 22 Network Office, Long Beach, California, United States of America; 4 Center for Information Technology, National Institutes of Health, Bethesda, Maryland, United States of America; 5 Laureate Institute for Brain Research and the University of Oklahoma College of Medicine, Tulsa, Oklahoma, United States of America; University of Oxford, United Kingdom

## Abstract

Anatomical differences in the corpus callosum have been found in various psychiatric disorders, but data on the genetic contributions to these differences have been limited. The current study used morphometric MRI data to assess the heritability of corpus callosum size and the genetic correlations among anatomical sub-regions of the corpus callosum among individuals with and without mood disorders. The corpus callosum (CC) was manually segmented at the mid-sagittal plane in 42 women (healthy, n = 14; major depressive disorder, n = 15; bipolar disorder, n = 13) and their 86 child or adolescent offspring. Four anatomical sub-regions (CC-genu, CC2, CC3 and CC-splenium) and total CC were measured and analyzed. Heritability and genetic correlations were estimated using a variance components method, with adjustment for age, sex, diagnosis, and diagnosis x age, where appropriate. Significant heritability was found for several CC sub-regions (P<0.01), with estimated values ranging from 48% (splenium) to 67% (total CC). There were strong and significant genetic correlations among most sub regions. Correlations between the genu and mid-body, between the genu and total corpus callosum, and between anterior and mid body were all >90%, but no significant genetic correlations were detected between ventral and rostral regions in this sample. Genetic factors play an important role in corpus callosum size among individuals. Distinct genetic factors seem to be involved in caudal and rostral regions, consistent with the divergent functional specialization of these brain areas.

## Introduction

The corpus callosum (CC) plays a major role in connecting the cerebral hemispheres, enabling inter-hemispheric communication [1]. The CC is the brain’s largest white matter structure [2], [3], and five anatomical sub-regions are commonly recognized: rostrum, genu, body, isthmus, and splenium [4]. The cortical origins of the axons passing through each subregion differ across sub regions. For example, projections from auditory and somatosensory cortices primarily traverse the body of the CC [5]–[7], while those from visual cortex traverse the splenium [5]–[7]. Further work is needed to characterize the functional roles of the inter-hemispheric projections contained within the CC, but the entire structure appears to play an integrative role [2] by facilitating the coordinated and rapid transfer of information involved in sensorimotor, attention, language, and other cognitive processes between contralateral, homologous cortical regions [7]–[10].

Disturbances in the structure or function of the CC have been reported in several neuropsychiatric illnesses, including schizophrenia [11]–[13], bipolar disorder [14], autism [15], and attention-deficit hyperactivity disorder [16]. In mood disorders, CC abnormalities have been implicated in some of the cognitive and other symptoms that occur in bipolar disorder [7], [17]; possibly reflecting compromised efficiency of information transfer between cerebral hemispheres [18]. Moreover, fractional anisotropy (a diffusion tensor imaging measure that reflects the degree to which white matter fibers are aligned in a specific direction) is significantly lower in the mid-body and genu of the CC in patients with bipolar disorder, compared to healthy controls [19], [20]. Abnormalities of size and morphology of the CC have also been reported in individuals with unipolar depressive disorders [21], [22], which may reflect alterations in the cortico-limbic-subcortical circuitry arising in association with the prefrontal cortical and temporal cortical volumetric differences reported in these conditions [23]–[25]. The relationship between CC structure and function under normal conditions, together with the evidence of structural alterations in mood disorders, underscores the importance of understanding the role of genes in influencing the size of the various substructures of the CC, and inter-relationships that reflect shared genetic factors.

Genetic factors have been shown to play important roles in the development of a variety of brain structures, including the CC. Several studies have shown that the volumes of specific brain structures are significantly heritable [25], [27]–[31]. For example, relatively high heritability estimates have been reported for cerebral hemisphere sizes [28], cortical morphology [32] and white matter volume [30]. Several investigations have reported significant heritability estimates for total CC size [32]–[34], although only a few study addressed the heritability of CC sub regions [35], [36]. While functional and anatomical connectivity between the CC and other brain regions (27] may imply shared genetic programs, there is a paucity of information regarding any genetic correlations among sub regions within the CC [38]. Thus it remains unclear how much of the morphologic correlation between sub-regions in the CC is attributable to shared genetic factors in humans.

The present study was aimed at investigating heritability of CC volume and genetic correlations among CC sub-regions in participants with and without mood disorders. The results demonstrate that genetic factors play an important role in CC volume, but also suggest that distinct genetic factors are involved in the development of caudal and rostral regions, consistent with the divergent functional specialization of these CC sub regions.

## Methods

### Ethics Statement

The participants provided their written informed consent. Written informed consent was obtained from the mothers on behalf of their children. The ethics committee of The National Institute of Mental Health (NIMH) approved this informed written consent procedure involving both adults and children. The ethics of conducting this experiment followed strictly the guidelines of The National Institutes of Mental Health (NIMH) Institutional Review Board (IRB), which examined and approved this research work.

### Participants

Participants were drawn from the NIMH Longitudinal Study of the children of affectively ill and well parents [39]. Forty-two mothers, ranging in age from 43 to 45 years, and 86 children (76 full siblings and 10 maternal half siblings) from 58 families underwent scanning, including 15 mothers with major depressive disorder (MDD), 13 with bipolar disorder (BD) and 14 with no psychiatric illness (control). All participants were in good physical health at enrollment and at time of scanning. Offspring included 33 from mothers with MDD, 25 from those with BD, and 28 from healthy mothers (Table 1). Offspring were initially enrolled between the ages of 1 and 3, classified by mother’s psychiatric status, and then reassessed periodically over the following 10-20 years. The MRI scans were acquired in offspring and mothers when offspring were between age 10 and 18 years. The original sample was largely of Caucasian ancestry, with a small representation of Hispanics, African Americans, and Asians (39). A previous study has shown that race did not have a significant effect on the various sub-regions of the corpus callosum [40]. Since heritability estimates in the present study depend on comparisons of mothers with their own offspring, racial differences between families have no influence on the estimates.

**Table 1:** Demographic data.

Mothers			Offspring			
			Males		Females	
Status	Number	Age	Number	Age (yrs.)	Number	Age (yrs.)
Control	14	45±5	15	15±3	13	16±2
Bipolar	13	43±4	9	15±3	16	15±2
Unipolar	15	45±6	13	15±2	20	17±2
Total	42		37		49	

### Magnetic Resonance Imaging

**Figure 1:**
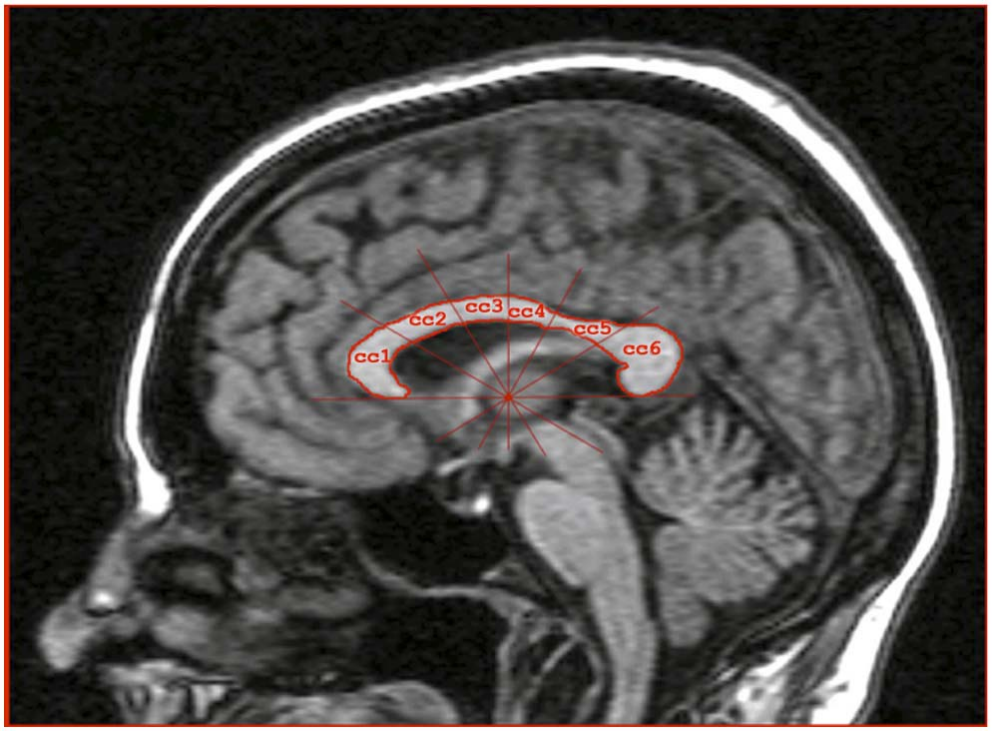
T1-weighted mid-sagittal view of the corpus callosum (CC). Six sub regions are shown: CC1 (genu), CC2, CC3, CC4, CC5 and CC6 (splenium).

Scans were obtained at the NIH using a Picker Vista 0.5 Tesla scanner running a gradient echo 3Dacquisition pulse sequence. Parameters for image acquisition were optimized for tissue contrast resolution in pilot studies performed in independent samples [41]. Sagittal images 2 mm thick were obtained at voxel size = 2×1×1 mm (TR = 20 ms, TE = 6 ms, flip angle = 45 degrees, FOV = 26 cm, matrix = 130×256, acquisition time = 7.9 14;minutes). Image data were processed with ANALYZE (Biomedical Imaging Resource, Mayo Foundation, Rochester, MN). In the mid-sagittal plane, the CC was manually traced and segmented into six sub regions, CC1 (corresponding to the relatively small rostrum plus the much larger genu region [42], CC2 (anterior CC body), CC3 (mid CC body), CC4 (posterior CC body), CC5 (isthmus), and CC6 (splenium) [40]. In the present study only the data from the CC1, CC2, CC3 and CC6 regions were analyzed, as these regions had the highest inter-rater reliability. Briefly, a horizontal line was drawn from the base of the splenium to the base of the genu. Vertical lines, perpendicular to the horizontal reference line, were drawn at the anterior aspect of the genu and posterior aspect of the splenium. The midpoint of the horizontal reference line between these two vertical reference lines were determined, and radial divisors were placed at that midpoint to divide the CC into sub-regions of C1, C2, C3 C4, C5 and C6 (Figure 1). Area measures (in mm^2^) were obtained from each sub-region and for the total CC area. Whole brain area was determined by manually tracing the cerebrum in the midsagittal plane. All tracings were performed with the rater blind to subject identity. Although this technology has now been replaced by higher resolution scanners, inter-rater reliability as measured by intra-class correlation [43] was excellent: 0.92 for the genu, 0.92 for the splenium, 0.90 for CC2, 0.90 for CC3 and 0.92 for total CC, based on 22 scans traced by two raters each. We did not have measures of overall brain size, but each sub region was expressed as a proportion of total CC size, thus minimizing the impact of individual differences in overall brain size.

### Quantitative Genetic Analysis

Information from full and half siblings and their mothers were used to conduct quantitative genetic analysis. The effects of age, sex, maternal psychiatric diagnosis, age^2^, and their interactions (age×sex, age×psychiatric diagnosis, sex×psychiatric diagnosis) were examined in a variance components analysis implemented by SOLAR [44]. Maternal diagnosis of MDD has been previously shown to affect CC size in offspring [45]. Covariates with significant statistical effects were included in the final model to determine heritability and genetic correlations.

Narrow-sense heritability (h^2^) and genetic correlations between brain regions were estimated using the maximum likelihood variance components method [44]. Narrow-sense heritability is defined as the proportion of the total phenotypic variance attributable to additive genetic variance:
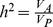



Genetic correlation analysis estimates the proportion of genetic variance that two traits share in common. A significant genetic correlation implies that a gene or genes shared among individuals influences both traits. A positive correlation indicates that genetic differences affect measured values in the same direction for both traits. SOLAR partitions the phenotypic variance into components attributable to genetic and environmental factors, and can account for the effects of covariates (age, sex, and diagnostic status).

Genetic correlations between CC volumes were investigated using a multivariate extension of the variance components method [43], [46]. This approach is based on the covariance between phenotypes of related individuals after accounting for the effects of significant covariates [47]. Likelihood methods were used to obtain the statistical significance of the estimate of each parameter by comparing a model in which that parameter is constrained to zero against the general model in which all parameters were estimated simultaneously. Further details regarding the population genetic theory and the specific analytical methods used can be found in Almasy and Blangero [46] and Comuzzie et al. [47].

## Results

### Estimation of Covariate Effects

In order to estimate the effects of potential confounding variables, age (including age^2^), sex, psychiatric diagnosis, and interactions were included as covariates in the initial model. Covariates that significantly influenced one or more CC subregions were included in the final model estimating heritability and genetic correlations (table 2).

**Table 2:** Significance levels of covariates and corresponding beta coefficients, by sub-regions of the corpus callosum.

	Traits									
Covariates	CC-genu		CC2		CC3		CC-splenium		CC-total	
	p-value	Beta	p-value	Beta	p-value	beta	p-value	beta	p-value	beta
Age	<0.001	0.04±0.0	<0.001	0.02±0.0	<0.001	0.03±0.0	<0.001	0.03±0.0	<0.001	0.03±0.0
Sex	ns	ns	<0.05	0.40±0.2	ns	ns	<0.001	0.67±0.2	<0.02	−.25±0.1
Diagnostic group	<0.01	0.27±0.1	ns	ns	ns	ns	<0.001	−.39±0.1	ns	0.52±0.2
Age x Diagnostic group	ns	ns	<0.05	−.01±0.0	ns	ns	<0.001	−.02±0.0	<0.02	−0.02±0.0

ns P>0.05.

Age significantly influenced size of the total CC and its sub regions (Table 2) while Age^2^ did not have a significant effect. Sex significantly influenced CC2, splenium (CC6) and total CC size, but did not significantly influence CC-genu or CC-3. Psychiatric diagnosis (MDD, BD or healthy) had a significant effect on the size of the genu, the splenium and CC-total but not on CC2 and CC3.

On the basis of these results, age was included as a covariate in all analyses, sex was included as a covariate for analyses of CC2, splenium, and total size, and diagnosis was included as a covariate for analyses of genu, splenium and CC-total. The interaction of age x diagnosis had a significant effect only on splenium and CC-total, hence it was included as a covariate in the analysis of splenium and CC-total.

### Heritability

Heritabilities were determined for each of the four CC sub-regions, along with the total.

CC size (Table 3). Significant heritability was found for the sizes of the genu, CC2, CC3, and total CC (all P<0.01), and for the splenium (P<0.05). Heritability (h^2^) values were substantial, he estimated heritability ranging from 48% (splenium) to 67% (total CC; Table 3). Thus genetic factors were found to have a substantial influence in all of the CC regions tested.

**Table 3:** Heritability (diagonal) and Genetic Correlations (above diagonal)^a^.

Area	CC-genu	CC2	CC3	CC-splenium	CC-Total
CC-genu	0.50±0.21**	0.71±0.20**	0.90±0.15**	0.85±0.16**	0.96±0.06**
CC2		0.62±0.22**	1.00±0.15**	0.54±0.24	0.87±0.10**
CC3			0.50±0.23**	0.58±0.27	0.96±0.07**
CC-splenium				0.48±0.22*	0.85±0.12**
CC-Total					0.67±0.22**

a Mean and standard deviation.

**P≤0.01,

*P≤0.05.

### Genetic Correlations

We found significant genetic correlations between all rostral and between all ventral sub regions (p<0.01), but not between CC2 and splenium or CC3 and splenium (Table 3). Particularly strong correlations were detected between the genu and mid-body structures (CC1 and CC3; 90%), between the genu and total CC (96%), and between anterior and mid-body structures (CC2 and CC3; 100%).

## Discussion

Brain size and morphology are significantly heritable [26]–[30]. Likewise, many previous studies have reported that the size of the corpus callosum (CC) is significantly heritable in humans [32]–[34], but few studies have characterized the heritability of CC substructures [60]. This is important, since anterior and rostral regions of the CC seem to perform distinct functions. Significant heritability estimates for area measures of the genu (66%), body (54%), and splenium (57%) have been reported [35]. In another paper, the same laboratory [36] reported significant heritabilities for genu (59%), body (62%), and splenium (75%). Significant heritabilities for CC substructures were also reported in non-human primates [48]. A recent genetic analysis of diffusion tensor images found significant heritability estimates for genu, body, and splenium [49].

The present study examined heritabilities and genetic correlations of total CC size and 4 sub-regions of the CC. While our sample size was limited, the heritability findings largely agree with previous reports, indicating that the size of the CC and its substructures are under strong genetic influence. We also show significant genetic correlations between many CC sub-regions. This conforms with the findings of Philips et al [48] in baboons, but has not to our knowledge been demonstrated before in humans. The present study also suggests for the first time that distinct genetic factors are involved in ventral and rostral regions of the CC. These results may have important implications for our understanding of how genetic factors operate during brain development.

We found significant, positive genetic correlations among all rostral, and among all ventral, sub regions of the CC, but not between ventral and rostral structures. While the genetic correlations between ventral and rostral structures are not significantly different from zero in this sample, only a larger sample could exclude the possibility that similar or overlapping genetic factors affect both ventral and rostral regions. Distinct genetic factors would be consistent with the divergent functional specialization of these brain regions [50]–[53]. Many genes have so far been shown to influence early brain development, but few studies have directly contrasted genetic influences on rostral and dorsal telencephalon. Among these, two patterning genes (Foxg1 and Fgf8) have been found to be involved in both rostral and dorsal telencephalic development in model organisms [54]–[56].

Most previous heritability studies of brain structure have used twin designs; the present study used parent-offspring pairs. Twin designs have many advantages, but also some pitfalls. Twins share similar environments from conception to birth and over the period during which they are reared together. This fact may confound shared genes with shared environments, inflating narrow sense heritability (h^2^) estimates [57]. Parent-offspring pair designs exclude shared prenatal environments, but do have other limitations, including shared recent environment, age differences, and uncertainties in offspring paternity. Shared current environment might be a major confound for certain traits, but brain anatomy is largely determined early in life, during which environmental influences on mothers and their offspring (typically born 20–30 years later) may be quite different.

Mothers in this sample were psychiatrically healthy or suffered from a major mood disorder. Despite this potentially important independent variable, psychiatric diagnosis had a significant effect only on the size of the genu and the splenium and did not significantly affect heritability estimates. Our results suggest that maternal bipolar and unipolar disorders do not substantially affect CC morphology – or do so similarly in mothers and their offspring. This finding contrasts with some previous work indicating that the genu of the corpus callosum was smaller in offspring of mothers with a history of MDD but not in the offspring of mothers with BD [45]. Our sample size did not permit comparisons between mothers with different types of mood disorder and we may have missed differences confined to MDD.

As in all imaging studies, the morphological differences we observed could represent cause or consequence of mood disorder. However, if mood disorders cause changes in CC morphology, this could not explain the significant correlation in CC size that we observed between mothers and their offspring. If on the other hand smaller CC contributes to mood disorder, then both CC size and mood disorder risk would be transmitted from mothers to offspring, consistent with our observations. Different study designs will be needed to fully disentangle the genetic influences on mood disorders from those on CC morphology.

We used ROI analyses to manually trace each brain area, blind to clinical and demographic variables. Some prior studies have suggested that this approach may be less sensitive to small variations in brain structure or tissue type distribution, compared to computer modeling methods [58]–[60]. On the other hand, manual tracing is not subject to many of the assumptions that underlie computer-modeling approaches.

In summary, our findings demonstrate that genetic factors play an important role in determining size of the CC and its substructures. While the same genes are involved in anatomically adjacent CC substructures, distinct genes seem to contribute to ventral and rostral regions. These findings suggest that the CC is a reasonable target for studies aimed at identifying the specific genes involved in regional brain development. To the extent that mental illnesses are disorders of brain circuitry, genes important in CC development are also likely to play an important role in mental illness, but this is still to be determined.

## References

[1] Schulte T, Muller-oehring EM (2010) Contribution of callosal connectors to the inter-hemispheric Integration of visuomotor and cognitive process. Neuropsychol Rev 20: 174–190. 20411431 10.1007/s11065-010-9130-1 PMC3442602

[2] Walterfang M, Malhi GS, Wood AG, Reutens DC, Chen J, Barton S, et al (2009) Corpus Callosum size and shape in established bipolar affective disorder. Aust NZJ Psychiatry 43: 838–845. 10.1080/00048670903107534 19670057

[3] Doron KW, Gazzaniga MS (2008) Neuroimaging techniques offer new perspectives on callosal transfer and interhemispheric communication. Cortex 44: 1023–1029. 18672233 10.1016/j.cortex.2008.03.007

[4] Aboitiz F, Scheibel AB, Fisher RS, Zaidel E (1992) Fiber composition of the human corpus callosum. Brain Res 598: 143–153. 1486477 10.1016/0006-8993(92)90178-c

[5] Hofer S, Frahm J (2006) Topography of the human corpus callosum revisited comprehensive fiber tractography using diffusion tensor magnetic resonance imaging. Neuroimage 32: 989–994. 16854598 10.1016/j.neuroimage.2006.05.044

[6] Risse G, Gates J, Lund G, Maxwell R, Rubens A (1989) Interhemispheric transfer in patients with incomplete section of the corpus callosum: anatomic verification with magnetic resonance imaging. Archive Neurol 46: 437–443. 10.1001/archneur.1989.00520400097026 2705905

[7] Gazzaniga MS (2000) Cerebral specialization and inter-hemispheric communication: Does the corpus callosum enable the human condition? Brain 123: 1293–1326. 10869045 10.1093/brain/123.7.1293

[8] Honey CJ, Thivierge J, Sporns O (2010) Can structure predict function in the human brain? Neuroimage 52: 766–776. 20116438 10.1016/j.neuroimage.2010.01.071

[9] Bruen PD, McGeown WJ, Shanks MF, Venneri A (2008) Neuroanatomical correlates of neuropsychiatric symptoms in Alzheimer’s disease. Brain 131: 2455–24633. 18669506 10.1093/brain/awn151

[10] Clarke J, Zaidel E (1994) Anatomical-behavioral relationships: corpus callosum morphometry and hemispheric specialization. Behav Brain Res 64: 185–202. 7840886 10.1016/0166-4328(94)90131-7

[11] Hauser P, Dauphinais DL, Berrettini W, De Lisi LE, Post RM (1989) Corpus callosum dimensions measured by magnetic resonance imaging in bipolar affective disorder and schizophrenia. Biologic Psychiatry 26: 659–668. 10.1016/0006-3223(89)90100-5 2804188

[12] Bachmann S, Pantel J, Flender A, Botter C, Essig M, et al (2003) Corpus callosum in first-episode patients with schizophrenia – a magnetic resonance imaging study. Psychol Med 33: 1019–102. 12946086 10.1017/s0033291703008043

[13] Price G, Bagary M, Cercignani M, Altman D, Ron M (2005) The corpus callosum in first episode schizophrenia: a diffusion tensor imaging study. J Neurol Neurosurg Psychiatry 76: 585–587. 15774453 10.1136/jnnp.2004.042952 PMC1739596

[14] Brambilla P, Barale F, Caverzasi E, Soares J (2004) Anatomical MRI findings in mood and anxiety disorders. Epidemiol Psichiatr Soc 11: 88–99. 10.1017/s1121189x00005558 12212470

[15] Vidal C, Nicolson R, DeVito T, Hayashi K, Geaga J, et al (2006) Mapping corpus callosum deficits in autism: an index of aberrant cortical connectivity. Biol Psychiatry 60: 218–225. 16460701 10.1016/j.biopsych.2005.11.011

[16] Yund G, Semrud G, Semrud-Clikeman M, Lorys A, Novey E, et al (1991) Corpus callosum morphology in attention deficit-hyperactivity disorder: morphometric analysis of MRI. J Learn. Disabil 24: 141–146. 10.1177/002221949102400302 2026955

[17] Soars JJ, Mann JC (1997a) The functional neuroanatomy of mood disorders. J Psychiatr Res 31: 393–432. 9352470 10.1016/s0022-3956(97)00016-2

[18] Schulte T, Sullian E, Muller-Oehring E, Adalsteinsson A, Pfefferbaum A (2005) Corpus callosal microstructureal integrity influences intermispheric processing: a diffusion tensor imaging study. Cereb Cortex 15: 1384–1392. 15635059 10.1093/cercor/bhi020

[19] Sussmann JE, Lymer GK, McKirdy J, Moorhead TW, Maniega SM, et al (2009) White matter abnormalities in bipolar disorder and schizophrenia detected using diffusion tensor magnetic resonance imaging. Bipolar Disord 11: 11–18. 19133962 10.1111/j.1399-5618.2008.00646.x

[20] Frazier JA, Breeze JL, Papadimitriou G, Kennedy DN, Hodge SM, et al (2007) White matter abnormalities in children with and at risk for bipolar disorder. Bipolar disord 9: 799–809. 18076529 10.1111/j.1399-5618.2007.00482.x

[21] Lacerda A, Brambilla P, Sassi R, Nicoletti M, Mallinger A, et al (2005) Anatomical MRI study of corpus callosum in unipolar depression. J Psychiatr Res 39: 347–354. 15804385 10.1016/j.jpsychires.2004.10.004

[22] Lyoo I, Kown J, Lee S, Han M, Chang C, et al (2002) Decrease in genu of the corpus callosum in medication-naïve, early-onset dysthymia and depressive personality disorder. Biol Psychiatry 52: 1134–1143. 12488058 10.1016/s0006-3223(02)01436-1

[23] Brambilla P, Barale F, Caverzasi E, Soares JJ (2002) Anatomical MRI findings in mood and anxiety disorders. Epidemiol Psychiatr Soc 11: 88–99. 10.1017/s1121189x00005558 12212470

[24] Soares JJ, Mann JC (1997b) The anatomy of mood disorders – review of structural neuroimaging studies. Biol. Psychiatry 41: 86–106. 10.1016/s0006-3223(96)00006-6 8988799

[25] Price JL, Drevets WC (2010) Neurocircuitry of mood disorders. Neurosychopharmacology 35: 192–216. 10.1038/npp.2009.104 PMC3055427 19693001

[26] Carmelli D, Swan GE, DeCarli C, Reed T (2002) Quantitative genetic modeling of regional brain volumes and cognitive performance in older male twins. Biol Psychol 61: 139–155. 12385673 10.1016/s0301-0511(02)00056-x

[27] Bartley AJ, Douglas Jones DW, Weinberger DR (1997) Genetic variability of human brain size and cortical gyral patterns. Brain 120: 257–269. 9117373 10.1093/brain/120.2.257

[28] Geschwind DH, Miller BL, DeCali C, Carmelli D (2002) Heritability of lobar brain volumes in twins supports genetic models of cerebral laterality and handedness. Proc Natl Acad Sci USA 99: 3176–3181. 11867730 10.1073/pnas.052494999 PMC122492

[29] Thompson PM, Cannon TD, Narr KL, Erp TV, Poutanen VP, et al (2001) Genetic influences on brain structure. Nat Neurosci 4: 1253. 11694885 10.1038/nn758

[30] Baare WFC, Pol HEH, Boomsma DI, Posthuma D, Geus EJ, et al (2002) Quantitative genetic modeling of variation in human brain morphology. Cereb Cortex 11: 816–824. 10.1093/cercor/11.9.816 11532887

[31] Todd RD, Bottern KN (2002) Etiology and genetics of early-onset of mood disorders. Child Adolesc Psychiatr Clin N Am 11: 499–518. 12222080 10.1016/s1056-4993(02)00013-5

[32] Hulshoff Pol HE, Schnack HG, Posthuma D, Mandl RC, Baare WFC, et al (2006) Genetic contributions to human brain morphology and intelligence. J Neurosci 26: 10235–10242. 17021179 10.1523/JNEUROSCI.1312-06.2006 PMC6674628

[33] Pfefferbaum A, Sullivan EV, Swan GE, Camelli D (2000) Brain structure in men remains highly heritable in the seventh and eighth decades of life. Neurobiology of aging 21: 63–74. 10794850 10.1016/s0197-4580(00)00086-5

[34] Scamvougeras A, Kigar DL, Jones D, Weinberger DR, Witelson SF (2003) Size of the human corpus callosum is genetically determined: an MRI study in mono and dizygotic twins. Neuroscience Letters 338: 91–94. 12566160 10.1016/s0304-3940(02)01333-2

[35] Kochunov P, Glahn DC, Lancaster J, Winkler A, Karlsgodt K, et al (2011) Blood pressure and cerebral white matter share common genetic factors in Mexican Americans. Hypertension 57: 3330–335. 10.1161/HYPERTENSIONAHA.110.162206 PMC3024472 21135356

[36] Kochunov P, Glahn DC, Lancaster JL, Winkler AM, Smith S, et al (2010) Genetics of microstructure of cerebral white matter using diffusion tensor imaging. NeuroImage 53: 1116. 10.1016/j.neuroimage.2010.01.078 PMC2888778 20117221

[37] Drevets WC, Price JL, Furey ML (2008) Brain structural and functional abnormalities in mood disorders: implications for neurocircuitary models of depression. Brain Struct Funct 213: 93–118. 18704495 10.1007/s00429-008-0189-x PMC2522333

[38] Badea A, Johnson GA, Williams RW (2009) Genetic dissection of the mouse brainusing high-field magnetic resonance microscopy. NeuroImage 45: 1067–1079. 19349225 10.1016/j.neuroimage.2009.01.021 PMC2667384

[39] Radke-Yarrow M, Nottelmann E, Martinez P, Fox M, Belmont B (1992) Young children of affectively ill parents: A longitudinal study of psychosocial development. Journal of American Academy of Child and Adolescence Psychiatry 31: 68–77. 10.1097/00004583-199201000-00011 1537784

[40] Lopez-Larson M, Breeze JL, Kennedy DN, Hodge SM, Tang L, et al (2010) Age related changes in the corpus callosum inearly-onset bipolar disorder assessed using volumetric and cross-sectional measurements. Brain imaging behave 4: 220–231. 10.1007/s11682-010-9101-4 PMC3711475 20686873

[41] Hauser P, Dauphinasis ID, Berrettini W, Delisi Le, Gelernter J, et al (1989) Corpus Callosium dimensions measured by magnetic response imaging in bipolar affective disorder and schizophrenia. Biol Psychiatry 26: 659–668. 2804188 10.1016/0006-3223(89)90100-5

[42] Witelson SF (1989) Hand and sex differences in the isthmus and genu of the human corpus callosum. A postmortem morphological study. Brain 112: 799–835. 2731030 10.1093/brain/112.3.799

[43] Hamer RM (2006) Compute six interclass correlation measures. SAS Documentation Aavailable: http://support.sas.com/cut/samples/index.jsp?sid=537&tab=details.

[44] Almasy L, Blango J (1998) Multpoint quantitative-trait linkage analysis in general pedigrees. Am J Hum Genet 62: 1198–1211. 9545414 10.1086/301844 PMC1377101

[45] Martinez P, Ronsaville D, Gold PW, Hauser P, Drevets WC (2002) Morphometric abnormalities in adolescent offspring of depressed mothers. Soc Neurosci Abstr 32.

[46] Almasy L, Blangero J (2009) Variance components methods for analysis of complex phenotypes. In: Almasy L, AL-Chalabi A. editors. Genetics of complex human diseases. Cold Spring Harbor, New York, 37–48. 10.1101/pdb.top77 PMC3064490 20439422

[47] Comuzzie AG, Rainwater DL, Blangero J Mahaney MC, VandeBerg JL, et al (1997) Shared and unique genetic effects among seven HDL phenotypes. Arterioscler Thromb Vasc Biol 17: 859–864. 9157948 10.1161/01.atv.17.5.859

[48] Phillips KA, Rogers J, Barrett EA, Glahn DC, Kochunov P (2012) Genetic contributions to the midsagittal area of the corpus callosum. Twin Res Human Genet 15: 315–323. 22856367 10.1017/thg.2012.10 PMC3474979

[49] Jahanshad N, Kochunov PV, Spooten E, Mandl RC, Nichols E, et al (2013) Multi-site genetic analysis of diffusion images and voxelwise heritability analysis: A pilot project of the ENIGMA-DTI working group. Neuroimage 81: 455–469. 23629049 10.1016/j.neuroimage.2013.04.061 PMC3729717

[50] Evans SJ, Choudary PV, Vawter MP, Li J, Meador-Woodruff JH, et al (2003) DNA microarray analysis of functionally discrete human brain regions reveals divergent transcriptional profiles. Neurobiology of disease 14: 240–250. 14572446 10.1016/s0969-9961(03)00126-8 PMC3098567

[51] Michael L, Anderson, Kinnison J, Luiz Pessoa L (2013) Describing functional diversity. of brain regions and brain networks. Neuroimage 73: 50–58. 23396162 10.1016/j.neuroimage.2013.01.071 PMC3756684

[52] Strenziok M, Greenwood PM, Santa Cruz SA, Thompson JC, Raja Parasuraman R (2013) Differential Contributions of Dorso-Ventral and Rostro-Caudal Prefrontal White Matter Tracts to Cognitive Control in Healthy Older Adults. Volume 8, Issue 12, e 81410, page2, 2013. 10.1371/journal.pone.0081410 PMC3846728 24312550

[53] Fanselow MS, Dong H (2010) Are the dorsal and ventral hippocampus functionally distinct structures?. Neuron 65: 7–19. 20152109 10.1016/j.neuron.2009.11.031 PMC2822727

[54] Storm EE, Garel S, Borello U, Hebert JM, Martinez S, et al (2006) Dose-. dependent functions of Fgf8 in regulating telencephalic patterning centers. Development 133: 1831–1844. 16613831 10.1242/dev.02324

[55] Martynoga B, Morrison H, Price DJ Mason JO (2005) Foxg1 is required for specification of. ventral telencephalon and region-specific regulation of dorsal telencephalic precursor. proliferation and apoptosis. Developmental Biology 283: 113–127. 15893304 10.1016/j.ydbio.2005.04.005

[56] Evans AE, Kelly CM, Precious SV, Rosser AE (2012) Molecular regulation of striatal. development: a Review. Hindawi publishing Corporation, Anatomy research International, Volume 2012, Article ID 106529, 14 pages. 10.1155/2012/106529 PMC3335634 22567304

[57] Falconer DS, Mackay TFC (1996) Introduction to quantitative genetics. Essex, England: Longman Group Limited. 160 p.

[58] Good CD, Ashburner J, Frackowiak RS (2001) Computational neuroanatomy: New. perspectives for neuroradiology Rev Neurol (Paris) 157: 797–806. 11677400

[59] Good CD, Scahill RI, Fox NC, Ashburner J, Friston KJ, e al (2002) Automatic. differentiation of anatomical patterns in the human brain: Validation with studies of degenerative dementias. Neuroimage 17: 29–46. 12482066 10.1006/nimg.2002.1202

[60] Fears SC, Service SK, Kremeyer B, Araya C, Araya X, et al (2014) Multisystem component phenotypes of bipolar disorder for genetic investigations of extended pedigrees. JAMA Psychiatry. 71: 375–87. 10.1001/jamapsychiatry.2013.4100 PMC4045237 24522887

